# Socio-economic, clinical and biological risk factors for mother - to – child transmission of HIV-1 in Muhima health centre (Rwanda): a prospective cohort study

**DOI:** 10.1186/0778-7367-71-4

**Published:** 2013-02-28

**Authors:** Maurice Bucagu, Jean de Dieu Bizimana, John Muganda, Claire Perrine Humblet

**Affiliations:** 1 World Health Organization Department of Maternal, Newborn, Child and Adolescent Health, 20, Avenue Appia, CH – 1211, Geneva 27, Switzerland; 2 CAMRIS International, 6931 Arlington Road, Suite 575, Bethesda, MD 20814, USA; 3 Department of Obstetrics & Gynecology, Rwanda Biomedical Center/ King Faisal Hospital, P.O. Box 2534, Kigali, Rwanda; 4 Université Libre de Bruxelles/Ecole de Santé Publique, Route de Lennik 808, 1070, Bruxelles, Belgium

**Keywords:** Socioeconomic, Clinical and biological risk factors, HIV-1, Mother - to – child transmission, Cohort, Muhima/Rwanda

## Abstract

**Background:**

Three decades since the first HIV-1 infected patients in Rwanda were identified in 1983; the Acquired Immunodeficiency Syndrome epidemic has had a devastating history and is still a major public health challenge in the country. This study was aimed at assessing socioeconomic, clinical and biological risk factors for mother – to – child transmission of HIV- in Muhima health centre (Kigali/Rwanda).

**Methods:**

The prospective cohort study was conducted at Muhima Health centre (Kigali/Rwanda).

During the study period (May 2007 – April 2010), of 8,669 pregnant women who attended antenatal visits and screened for HIV-1, 736 tested HIV-1 positive and among them 700 were eligible study participants. Hemoglobin, CD4 count and viral load tests were performed for participant mothers and HIV-1 testing using DNA PCR technique for infants.

Follow up data for eligible mother-infant pairs were obtained from women themselves and log books in Muhima health centre and maternity, using a structured questionnaire.

Predictors of mother-to-child transmission of HIV-1 were assessed by multivariable logistic regression analysis.

**Results:**

Among the 679 exposed and followed-up infants, HIV-1 status was significantly associated with disclosure of HIV status to partner both at 6 weeks of age (non-disclosure of HIV status, adjusted odds ratio [AOR] 4.68, CI 1.39 to 15.77, p < 0.05; compared to disclosure) and at 6 months of age (non-disclosure of HIV status, AOR, 3.41, CI 1.09 to 10.65, p < 0.05, compared to disclosure).

A significant association between mother’s viral load (HIV-1 RNA) and infant HIV-1 status was found both at 6 weeks of age (> = 1000 copies/ml, AOR 7.30, CI 2.65 to 20.08, p < 0.01, compared to <1000 copies/ml) and at 6 months of age (> = 1000 copies/ml, AOR 4.60, CI 1.84 to 11.49, p < 0.01, compared to <1000 copies/ml).

**Conclusion:**

In this study, the most relevant factors independently associated with increased risk of mother – to – child transmission of HIV-1 included non-disclosure of HIV status to partner and high HIV-1 RNA. Members of this cohort also showed socioeconomic inequalities, with unmarried status carrying higher risk of undisclosed HIV status. The monitoring of maternal HIV-1 RNA level might be considered as a routinely used test to assess the risk of transmission with the goal of achieving viral suppression as critical for elimination of pediatric HIV, particularly in breastfeeding populations.

## Background

Three decades since the first HIV-1 infected patients in Rwanda were identified (1983), the Acquired Immunodeficiency Syndrome (AIDS) epidemic has had a devastating history and is still a major public health challenge in country [1,2].

At the end of 2010, an estimated 34 million people [31.6 million-35.2 million] were living with HIV worldwide, up 17% from 2001. The proportion of women among people living with HIV has remained stable at 50% globally, but they are more affected in Sub-Saharan Africa (59% of all people living with HIV). Mother-to-child transmission of HIV remains the primary mode of child contamination during pregnancy, childbirth or breastfeeding. It is estimated that every day there are over 1,000 new HIV infections in children, with vast majority occurring in Sub-Saharan Africa. Nearly 370,000 [230,000 - 510,000] children were infected with HIV through mother- to- child transmission globally in 2009. The scaling up of effective interventions for the prevention of HIV transmission from mother- to- child (PMTCT) is still limited because of inadequate access to antenatal and postnatal services, particularly in developing countries [3].

With a population of 10.4 million (2010), of whom majority are female (52%), young (67% have less than 25 years) and living in rural areas (83%), Rwanda faces enormous challenges of political, social, economic, health development, and particularly those relating to the consequences of the 1994 genocide. Its gross domestic product is estimated at USD 540 $/capita (2010), with a high level of poverty (56.9% below the poverty line, 2005) [4]. HIV trend from Rwanda Demographic and Health surveys data shows that adult HIV prevalence has remained unchanged from 2005 to 2010. Estimated nationally at 3%, 4% in women and 2% in men, it is 8.7% in urban areas and 2.8% in rural areas (2010) [5].

The programme for prevention of mother-to-child transmission of HIV-1 was put in place in 1999, with a goal of reducing the incidence of new HIV infections among children born to HIV positive mothers, with the use of antiretroviral medicines [6,7]. The package of PMTCT services provided during the study period (2007–2010) included antenatal care with testing and counseling; essential obstetric and neonatal care; triple therapy and dual therapy ARV respectively for HIV-positive mothers and infants; as well as family planning [8]. Country’s commitments, towards eliminating new HIV infections among children by 2015 and keeping their mothers alive, are in line with the global plan launched by the UN Secretary General in 2011. The following specific targets were defined at global level: overall transmission rate < 5% at the population level (<2% in the absence of breastfeeding or measured at 6 weeks) and the reduction of new pediatric HIV infections by 90% from the estimated baseline [9].

In developed countries, the rate of HIV transmission from the mother to the child has been significantly reduced to 1%, through the use of antiretroviral triple therapy for HIV-infected mother, caesarean delivery and formula feeding for the child. In Rwanda, the mother-to-child transmission of HIV-1 before the era of antiretroviral medicines, has affected more than one in four children (25.7%) born to HIV-1 positive mothers [2].

During the decade of 2000s, the use of antiretroviral drugs allowed for significant advance in the reduction of HIV mother-to-child transmission. However, feeding practices for exposed children still represent a major challenge for populations with breastfeeding as the only feeding alternative for child survival. Nearly all Rwandan children are breastfed through the first year of life, with about 70% of them receiving complementary foods by age of 6 to 9 months [5].

Several studies support the role of socioeconomic, clinical and biological risk factors for HIV-1 transmission [8-12]. Based on this background, we initiated this study to assess the role of socioeconomic, clinical and biological factors in mother-to-child transmission of HIV-1 at Muhima health centre (Kigali/Rwanda), where this type of study has never been conducted. The site of the study includes Muhima health centre & hospital, located in the district of Nyarugenge (Kigali), with a population of about 287,529 inhabitants (2010).

This study is specifically expected to allow for better understanding of risk factors, likely to limit the effectiveness of the national strategy for elimination of mother-to-child transmission of HIV-1. The knowledge generated from this study in Muhima will guide the national evidence-based response towards a multisectoral approach, with both more effective health interventions and specific socioeconomic strategies to address the HIV pandemic in Rwanda [1,9,13].

## Methods

### Study design and population

The prospective cohort study was conducted at Muhima health centre (Kigali/Rwanda).

All pregnant women diagnosed with HIV-1 and attending PMTCT service at Muhima health centre were invited to participate in the study, between May 2007 and April 2010. Eligible study participants were pregnant HIV-1 infected women, consenting to the study, who had attended antenatal visits or delivered at Muhima maternity and had benefited from PMTCT interventions in line with the national guidelines (based on combination of Zidovudine/Lamivudine/Nevirapine for mothers during pregnancy, childbirth and postnatal period and Nevirapine/Zidovudine for newborn). Additional inclusion criteria was for participants to be registered as residents within the specific catchment area of Muhima health centre and therefore expected to attend the postnatal follow up as required. All HIV negative pregnant women, those whose consent to the study was not obtained and those living outside the catchment area of Muhima were excluded from the study.

We estimated the sample size based on anticipated HIV-1 infection of 4% at 6 weeks and absolute precision in% points of 1.5, with a confidence interval (CI) of 95%. A sample size of 656 was the minimum number required for the study. During the study period, of 8,669 pregnant women who attended antenatal visits and screened for HIV-1 in Muhima health centre, 736 tested HIV-1 positive and among them 700 were eligible study participants [14]. At enrolment, eligible participants were interviewed by three trained PMTCT providers (2 data collectors supervised by 1 medical doctor) until the determined sample size of 700 women was reached. Information was collected from each mother – infant pair for a period of 6 months after childbirth, including specific socioeconomic characteristics, clinical and biological features. For twins, one member randomly selected from each twin pair was included in the study.

### Data collection and management

Follow up data for eligible mother-infant pairs, about pregnancy, childbirth and postnatal period were obtained from women themselves and log books in Muhima health centre and maternity, using a structured questionnaire, translated into Kinyarwanda by the principal investigator. Those data included medical records and laboratory tests results. As viral load was not a requirement for the national PMTCT protocol, it was performed by the Rwanda National Referral laboratory for study participants at the request of the author, using COBAS TaqMan HIV-1 test or Amplicor HIV-1 Monitor test v1.5 (both from Roche Diagnostic Corporation, Indianapolis, IN). This is nucleic acid amplification test for the quantification of Human Immunodeficiency Virus Type 1 RNA in human plasma.

Required data were collected anonymously, using participant’s unique identifier, nationally provided by the National Centre for Treatment and Research on AIDS, Malaria, Tuberculosis and other epidemics (Rwanda TRAC plus/Rwanda Ministry of Health).

The study was designed to allow for periodic re-questioning of study participants, at birth, 6 weeks and 7 months postpartum, 1 month after cessation of breastfeeding as per the national guidelines [1].

Twenty –one study participants were considered lost to follow up as they have not shown up for regular visits and the study team unable to find HIV-1 tests results for their infants at 6 weeks and/or at 6 months (Dried Blood Spot method using PCR technique). Baseline data on known variables, namely age, marital status, maternal education, residence, wealth index were found to be sufficiently similar to those of participants (679) who remained in the study [15,16].

The data were double entered for all 700 questionnaires, by a team of 2 data entry clerks supervised by a lecturer from the University of Rwanda/School of Public Health.

Data quality assessment was performed in two steps: A review of all the records for participants with caesarean section as mode of delivery (121 cases) was conducted: 90% of self-reports were confirmed by relevant medical records. Information on mode of delivery, in this study, was provided by the participant and confirmed with the maternal health card. The quality control was meant for checking data consistency between the study questionnaires and medical records from the health facility where childbirth took place. For 12 participants in the study (i.e. 10%), expected data were not found in the Muhima hospital register. The missing medical records were later found in a logbook that was kept in the Kigali University Hospital (CHUK). The logbook was taken by the CHUK team, when it went back to its premises after rehabilitation (2009) and temporary relocation in Muhima hospital (the study site) in 2007–2008.

In addition, all viral load tests results for 679 study participants were jointly verified by the principal investigator and the laboratory manager, before the required payment could be executed by Muhima hospital.

The main outcome was cumulative incidence of mother – to – child transmission of HIV-1. It was measured at 6 weeks and 6 months of life among live born children [17,18].

### Data analysis

Background data were summarized with descriptive statistics. Univariate analyses of associations were performed using the chi squared test, Fisher’s test as appropriate. Potential risk factors for mother – to – child transmission of HIV-1 were considered as covariates, including: socioeconomic characteristics that included mother’s age in years (< =24 years/> 24 years); marital status (married/unmarried); mother’s education (no education/primary education; secondary school and more); parity (primiparous/multiparous); wealth index re-categorized in 5 quintiles (poorest/second/middle/fourth/richest) (Demographic Health Survey wealth index model); disclosed HIV status to partner (yes/no); sex of infant (male, female), infant feeding choices during the first 6 months of life (exclusive breastfeeding/artificial feeding/mixed feeding) [19].

Clinical factors were: mode of delivery (vaginal/instrument assisted/cesarean section); type of ARV treatment (prophylactic/curative); duration of ARV treatment prior to delivery (< 6 weeks/> = 6 weeks).

Biological factors included hemoglobin during pregnancy (<11 g/dl/> = 11 g/dl); CD4 count (< 350 cells/μL/> = 350 cells/μL); viral load (HIV-1 RNA) (< 1000 copies/mL/> = 1.000 copies/mL).

Predictors of mother-to-child transmission of HIV-1 were assessed by multivariable logistic regression. All variables with potential association with the main outcome – HIV-1 transmission, were entered into the logistic regression model. Hosmer and Lemeshow test was applied to check for how well the model fit. Variables were held in the model if they reached a significance level of P < 0.05.

**Figure 1:**
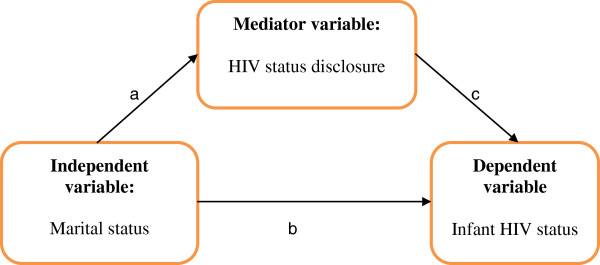
**Path diagram for mediation effect of HIV status disclosure.** Mothers and infants meeting inclusion criteria in the study. Muhima health centre cohort, Kigali/Rwanda, 2007-2010 [21]. The following steps were tested, as applied in social psychological research: first the independent variable affects the mediator in the first equation (**a**); second, the independent variable affects the dependent variable in the second equation (**b**); and third, the mediator affects the dependent variable in the third equation (**c**).

In addition, to demonstrate the potential mediation effect of HIV disclosure variable through which a focal independent variable (marital status) is able to influence the dependent variable (infant HIV status), as shown in Figure 1, the following steps were tested, as applied in social psychological research: first the independent variable affects the mediator in the first equation; second, the independent variable affects the dependent variable in the second equation; and third, the mediator affects the dependent variable in the third equation.

A series of regression models was used to quantify the relationships between marital status and HIV status disclosure (a); marital status and infant HIV status (b), HIV status disclosure and infant HIV status (c) [20-23].

Statistical analyses were performed with STATA 12.0 (Stata Corp, College Station, TX) and Statistical Package for the Social Sciences (SPSS) version 17.

### Ethical considerations

The study protocol was reviewed and approved by the Rwanda national ethical committee and the research commission of University Teaching Hospital of Kigali, in February 2007, with annual evaluation of the study progress. An informed consent has been obtained, with a written and signed document, for each participant in this cohort study.

## Results

### Baseline characteristics

Socioeconomic, clinical and biological characteristics of mother-infant pairs enrolled in the study are shown in Table 1. Nearly 1 in 3 mothers (29.7%) were young, with a median age of 27 years (range: 17 – 45 years). Majority of mothers had only primary or no education (74.7%) and were married (82.7%). Two in five (40.3%) study participants were classified by wealth index quintiles as poorest and poor. Over one fifth (21.5%) of women were primiparas, with median number of 2 births (range: 1–9 births). For 18.9% of women, male partner’s HIV status was not disclosed. Only 2.9% of babies were born at home; for 17.8% of pregnant women, babies were delivered by cesarean section. Over half (50.7%) of exposed infants were females. The vast majority (86.1%) of mothers reported that their children received exclusive breastfeeding. At enrollment, the median hemoglobin was 12.20 g/dl (range: 6.30 – 19 g/dl); with median CD4 count of 429.50 cells/mm^3^ (range: 11 – 1718 cells/mm^3^) and median viral load of 40 copies/ml (range: 40 – 890,000 copies/ml); 63.3% of participant mothers had a viral load of less than 40 copies/ml. Of 679 mothers, majority (51.8%) reported they have received HIV chemoprophylaxis regimen and 77.5% have taken ARVs for at least 6 weeks prior to childbirth.

**Table 1:** Background characteristics of mothers and infants meeting inclusion criteria in the study

**Variable**	**Number (N = 679)**	**Percentage**
**Mother’s age in years**		
<=24	202	29.7
>24	477	70.3
**Marital status**		
married	562	82.7
unmarried	117	17.3
**Mother’s education**		
none/primary	509	75.0
secondary/university	170	25.0
**Wealth**		
poorest	175	25.8
poor	99	14.5
middle	143	21.1
rich	145	21.4
richest	117	17.2
**Parity**		
primipara	146	21.5
multiparous	533	78.5
**Disclosed HIV status to partner**		
Yes	551	81.1
No	128	18.9
**Place of delivery**		
home	20	2.9
facility	659	97.1
**Sex of infant**		
male	335	49.3
female	344	50.7
**Type of feeding during first 6 months of life**		
exclusive breastfeeding	585	86.1
artificial feeding	80	11.8
mixed feeding	14	2.1
**Mode of delivery**		
vaginal/instrument assisted	558	82.2
cesarean section	121	17.8
**Hemoglobin during pregnancy**		
<11 g/dl	180	26.5
> = 11 g/dl	499	73.5
**Type of ARV treatment**		
prophylactic	352	51.8
curative	327	48.2
**Duration of ARV treatment prior to delivery**		
less than 6 weeks	153	22.5
6 weeks or more	526	77.5
**CD4 count**		
<350 cells/mm^3^	263	38.7
> = 350 cells/mm^3^	416	61.3
**Viral load**		
<1000 copies/ml	517	76.1
> = 1000 copies/ml	162	23.9

Muhima health centre cohort, Kigali/Rwanda, 2007 – 2010.

### Infant HIV-1 infection

**Figure 2:**
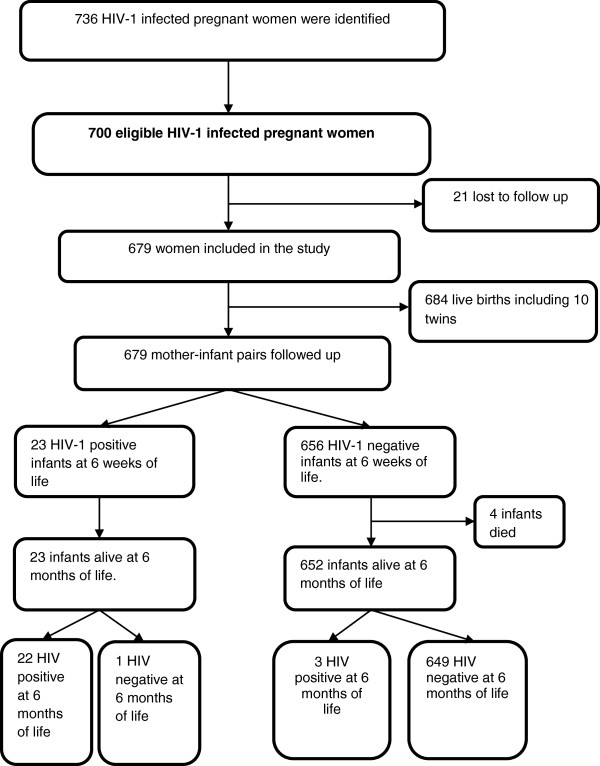
**Muhima health centre cohort profile: mothers and infants meeting inclusion criteria in the study.** Kigali/Rwanda, 2007-2010.

The study enrolled a cohort of 700 HIV-1 infected pregnant women and their newborn. Of these 21 study participants were lost to follow up and 679 pregnant women had live births of 674 single babies and 10 twins (Figure 2). At 6 weeks, of the 679 live born babies and followed up, a total of 23 infants tested HIV-1 positive. Among these, at 6 months, 22 infants were confirmed HIV-1 infected and 1 infant uninfected with HIV. Among the 656 initially HIV-negative infants, 4 died before 6 months of age (with no confirmed diagnosis). Of the 652 remaining infants, 3 were confirmed HIV-1 infected between 6 weeks and 6 months of age (11.5% of all infected infants). The overall HIV-1 cumulative incidence of mother – to – child transmission rate was 3.7% at 6 weeks to 6 months of age and 3.2% was at zero to 6 weeks of age.

### Risk factors for mother-to-child transmission of HIV-1

In univariate analysis, among the 679 HIV exposed live infants, there is a statistically significant association between HIV-1 status of infants and their mothers’ marital status; parity; hemoglobin level, duration of ARV treatment, CD4 count and viral load, as well as HIV status disclosure to partner and infant feeding choices. Variables not associated with HIV-1 status of infants included mother’s age, mother’s education, wealth, mode and place of childbirth and type of ARV treatment (Table 2).

**Table 2:** Univariate analysis of factors associated with mother-to-child transmission of HIV-1 at 6 weeks and at 6 months of life

**Variables**	**Infant HIV-1 status at 6 weeks**				**Infant HIV-1 status at 6 months**			
	**n**	**HIV-(%)**	**HIV + (%)**	**P-value**	**N**	**HIV-(%)**	**HIV + (%)**	**P-value**
**Mother’s age, years**								
<=24	202	95.5	4.5	0.317	202	94.6	5.4	0.117
>24	477	97.1	2.9		473	97.0	3.0	
**Marital status**								
married	562	97.7	2.3	0.001	559	97.5	2.5	0.000
unmarried	117	91.5	8.5		116	90.5	9.5	
**Education**								
none/primary	509	96.5	3.5	0.710	508	96.3	3.7	0.930
secondary/university	170	97.1	2.9		167	96.4	3.6	
**Wealth**								
poorest	175	94.3	5.7	0.328	174	94.3	5.7	0.459
second	99	98.0	2.0		99	98.0	2.0	
middle	143	97.2	2.8		143	97.2	2.8	
fourth	145	96.6	3.4		144	95.8	4.2	
richest	117	98.3	1.7		115	97.4	2.6	
**Parity**								
primipara	146	93.2	6.8	0.009	144	91.7	8.3	0.001
multiparous	533	97.6	2.4		531	97.6	2.4	
**Disclosed HIV status to partner**								
Yes	551	98.2	1.8	0.000	550	97.8	2.2	0.000
No	128	89.8	10.2		125	89.6	10.4	
**Place of delivery**								
home	20	90.0	10.0	0.097	20	90.0	10.0	0.130
facility	659	96.8	3.2		655	96.5	3.5	
**Sex of the infant**								
male	335	96.7	3.3	0.883	332	96.4	3.6	0.904
female	344	96.5	3.5		343	96.2	3.8	
**Type of feeding during first 6 months of life**								
exclusive breastfeeding	585	96.8	3.2	0.070	581	96.6	3.4	0.002
artificial feeding	80	97.5	2.5		80	97.5	2.5	
mixt feeding	14	85.7	14.3		14	78.6	21.4	
**Mode of delivery**								
vaginal/instrument assisted	558	96.4	3.6	0.542	554	96.0	4.0	0.431
cesarean section	121	97.5	2.5		121	97.5	2.5	
**Hemoglobin during pregnancy**								
<11 g/dl	180	93.3	6.7	0.005	179	93.9	6.1	0.044
> = 11 g/dl	499	97.8	2.2		496	97.2	2.8	
**Type of ARV treatment**								
prophylactic	352	97.4	2.6	0.215	350	96.9	3.1	0.423
curative	327	95.7	4.3		325	95.7	4.3	
**Duration of ARV treatment prior to delivery**								
less than 6 weeks	153	93.5	6.5	0.014	152	94.1	5.9	0.100
6 weeks or more	526	97.5	2.5		523	96.9	3.1	
**CD4 count**								
<350 cells/mm^3^	263	94.3	5.7	0.009	261	94.3	5.7	0.028
> = 350 cells/mm^3^	416	98.1	1.9		414	97.6	2.4	
**Viral load**								
<1000 copies/ml	517	98.6	1.4	0.000	514	98.1	1.9	0.000
> = 1000 copies/ml	162	90.1	9.9		161	90.7	9.3	

Muhima health centre cohort, Kigali/Rwanda, 2007 – 2010.

In a multivariable logistic regression analysis (Table 3), the most relevant variables that remained significant in the model included disclosure of HIV status to partner and viral load (HIV-1 RNA).

**Table 3:** Multivariable logistic regression analysis of risk factors associated with mother-to-child transmission of HIV at 6 weeks and at 6 months of life

	**HIV infection at 6 weeks**	**HIV infection at 6 months**
	**(1)**	**(2)**
**Variables**	**adjusted OR [95% CI]**	**adjusted OR [95% CI]**
**Mother’s age**		
<=24	1.22 [0.41 - 3.64]	1.43 [0.53 - 3.89]
>24	1	1
**Marital status**		
not currently married	1.42 [0.42 - 4.79]	1.55 [0.49 - 4.90]
currently married	1	1
**Education attainment**		
none/primary	1.09 [0.29 - 4.08]	1.09 [0.33 - 3.63]
secondary/university education	1	1
**Wealth index**		
poorest	2.07 [0.31 - 14.01]	1.37 [0.27 - 7.01]
second	0.77 [0.07 - 8.16]	0.39 [0.05 - 3.44]
middle	0.82 [0.11 - 5.90]	0.53 [0.09 - 2.94]
fourth	1.33 [0.21 - 8.32]	1.05 [0.23 - 4.91]
richest	1	1
**Parity**		
primiparous	1.97 [0.65 - 5.94]	2.33 [0.84 - 6.41]
multiparous	1	1
**Disclosed HIV status to partner**		
No	4.68** [1.39 - 15.77]	3.41** [1.09 - 10.65]
Yes	1	1
**Place of delivery**		
home	2.98 [0.42 - 21.17]	2.21 [0.33 - 14.88]
health facility	1	1
**child’s sex**		
Male	1.27 [0.49 - 3.34]	1.21 [0.49 - 2.98]
Female	1	1
**Type of feeding for the child during first 6 months of life**		
mixed feeding	5.5 [0.40 - 74.82]	9.64* [0.96 - 96.62]
artificial feeding	2.45 [0.39 - 15.24]	1.99 [0.36 - 11.10]
exclusive breastfeeding	1	1
**Mode of delivery**		
vaginal/instrument assisted	0.66 [0.17 - 2.57]	0.91 [0.24 - 3.40]
cesarean section	1	1
**Hemoglobin**		
<11 g/dl	2.58* [0.90 - 7.40]	1.73 [0.65 - 4.62]
> = 11 g/dl	1	1
**Type of ARV treatment received**		
prophylactic	1.82 [0.38 - 8.73]	1.79 [0.43 - 7.41]
curative	1	1
**duration of ARV prophylaxis/treatment**		
less than 6 weeks	2.24 [0.81 - 6.16]	1.35 [0.51 - 3.57]
6 weeks or more	1	1
**CD4 count**		
<350 cellules/mm^3^	4.83* [0.98 - 23.90]	3.82* [0.92 - 15.94]
> = 350 cellules/mm^3^	1	1
**Viral load**		
> = 1000 copies/ml	7.30*** [2.65 - 20.08]	4.60*** [1.84 - 11.49]
<1000 copies/ml	1	1
Observations	679	675

*** p < 0.01, ** p < 0.05, * p < 0.1.

The following variables were considered for inclusion in the logistic regression model: mother’s age; marital status; wealth; parity; HIV status disclosure to partner; place of delivery; mode of delivery; child sex; infant feeding choice; hemoglobin; type of ARV treatment received; duration of ARV prophylaxis/treatment; CD4 count and viral load (HIV-1 RNA).

Muhima health centre cohort, Kigali/Rwanda, 2007 – 2010.

Among exposed infants, HIV-1 status was significantly associated with disclosure of HIV status to partner both at 6 weeks of age (non-disclosure of HIV status, adjusted odds ratio [AOR] 4.68, CI 1.39 to 15.77, p < 0.05; compared to disclosure) and at 6 months of age (non-disclosure of HIV status, AOR, 3.41, CI 1.09 to 10.65, p < 0.05, compared to disclosure).

A significant association between mother’s viral load (HIV-1 RNA) and infant HIV-1 status was found both at 6 weeks of age (> = 1000 copies/ml, AOR 7.30, CI 2.65 to 20.08, p < 0.01, compared to <1000 copies/ml) and at 6 months of age (> = 1000 copies/ml, AOR 4.60, CI 1.84 to 11.49, p < 0.01, compared to <1000 copies/ml).

In the study multivariable model, the following covariates were found with limited statistical significance: infant feeding choice at 6 months of age (mixed feeding, AOR 9.64, CI 0.96 to 96.62, p < 0.1, compared to exclusive breastfeeding); mother’s CD4 count both at 6 weeks of infant age (<350 cells/mm^3^, AOR 4.83, CI 0.98 to 23.90, p < 0.1, compared to > =350 cells/mm^3^) and at 6 months of infant age (<350 cells/mm^3^, AOR 3.82, CI 0.92 to 15.94, p < 0.1, compared to > =350 cells/mm^3^); mother’s hemoglobin level at 6 weeks of infant age (<11 g/dl**,** AOR 2.58, CI 0.90 to 7.40, p < 0.1, compared to > =11 g/dl).

## Discussion

This study assessed socioeconomic, clinical and biological risk factors for mother – to – child transmission of HIV-1 among 679 infants (at 6 weeks) and 675 (at 6 months) born to HIV infected mothers and followed up at Muhima health centre (Kigali/Rwanda).

This vertical transmission occurs at three stages including prepartum, intrapartum and postpartum (breastfeeding) [24-26].

In the Muhima cohort study, were most at risk of mother – to – child transmission, HIV-1 exposed infants whose mothers presented with no documented mutual disclosure of HIV status and a higher HIV-1 RNA level.

### Mutual disclosure of HIV status

Consistent with other studies, non-disclosure of HIV status to partner emerged as an important factor for HIV-1 mother – to – child transmission in this cohort study.

Existing studies have increasingly shown disclosure as a way to encourage prevention and non-disclosure significantly associated with sexual risk behaviours.

Disclosure is of importance in PMTCT programmes as it allows an individual to get spousal or family support for preventive actions they may decide to undertake, including approaches for adequate ARV adherence [27-31].

Fear of stigma and rejection is thought to discourage disclosure [32].

Women appear to disclose, and to receive disclosure, more frequently than do men. Partner disclosure is also generally lower with casual partners than it is with steady partners.

A positive correlation between disclosure and social support has been documented in various contexts. Socioeconomic factors (education) and access to resources play an important role in influencing disclosure, with lower rates of disclosure associated with low-wage employment and economic vulnerability [33].

In Zimbabwe, receiving HIV education during antenatal visits at least twice and referral for psychosocial support were significantly protective [34] In Rwanda, a package of strategies – including couple counseling and community campaigns – seems to have overcome some barriers to disclosure [32].

From the social psychological analysis applied in this study, the required four conditions allowing for testing the validity of the mediation model were satisfied (Figure 1) [20,23]. This is considered as a perfect mediation model as the independent variable (marital status) has shown no effect on the dependent variable (infant HIV-1 status) when the mediator is controlled (HIV status disclosure). Unmarried women infected with HIV-1 have higher risk of HIV-1 mother-to-child transmission with undisclosed HIV status, as a mediator. The mediator variable explains how external physical events (independent variable) take on internal psychological significance [21-23].

### Viral load

The level of HIV-1 RNA in maternal plasma remains the major biological predictor of both early and late mother-to-child transmission of the virus. HIV-infected individuals who maintain increased levels of HIV-1 RNA load, extended high viremics, can transmit virus at higher rates. Combinatorial ART decreases HIV replication, thus reducing rates of virus transmission [35,36].

In addition, existing studies have shown that, for women receiving HAART for prevention of HIV-1 transmission, breast milk HIV-1 RNA level is associated with systemic viral burden [37].

The total viral suppression (below 40 copies/mL for this study) provides the most protective effect against mother-to-child transmission of HIV-1 [38,39].

Achieving viral suppression by delivery to prevent MTCT depends on maternal viral load baseline level and time of HAART initiation during pregnancy as shown by a UK study findings. This suggest that with a viral load of more than 10,000 copies/mL and especially with a viral load more than 100,000 copies/mL, the probability of achieving either less than 50 copies/mL by the time of delivery is compromised by delaying initiation of short-term highly active antiretroviral therapy beyond 20.4 weeks gestation [40].

### Breastfeeding

Like many studies investigating feeding choices for HIV-1 exposed infants in breastfeeding populations, the Muhima cohort study results show that exclusive breastfeeding was associated with a lower risk of postnatal transmission at 6 months, compared with mixed feeding, but it does not eliminate risk. The only method known to completely eliminate breastfeeding associated HIV transmission is not to breastfeed. This is recommended in settings in which infant replacement feeding is affordable and sustainable [41-44].

A prospective study in Zimbabwe with 14,110 mother-newborn pairs followed for 2 years showed that early mixed feeding, when compared with early breastfeeding, was associated with a 2.5 fold (95CI 1.3 to 4.8) greater risk of HIV infection or death at 18 months [45].

Breast milk protective mechanisms include factors that have the ability to inactivate HIV and/or binding to the infant mucosa and/or target cells. Milk also contains many anti-inflammatory factors that would limit viral replication within milk, as well as maintain the integrity of both the mammary (reducing transmissibility) and the infant mucosal epithelia (reducing susceptibility) [46].

The WHO infant feeding guidelines in the context of HIV have been revised to ensure balance between HIV prevention with protection from other causes of child mortality. They recommend that national or subnational authorities should decide whether health services will principally counsel and support HIV infected mothers to either avoid all breastfeeding or to breastfeed and receive infant or maternal antiretroviral prophylaxis [43].

In Rwandan general population, exclusive breastfeeding is the norm. As reported by Rwanda Demographic and Health Survey 2010 results, eighty-five percent of children under age of 6 months are exclusively breastfed, 2 percent are given milk and plain water, 7 percent get breast milk and non-milk liquids, and 3 percent take other types of milk in addition to breast milk [6]. With regard to mixed feeding in the context of HIV/AIDS in Rwanda, an evaluation of infant feeding and young child feeding practices reported high prevalence of exclusive breastfeeding with weaning at 4–6 months. The author found no indication that mothers who mixed fed did so because they believed their breast milk was insufficient. This finding suggest that it should be possible to achieve even higher rates of exclusive breastfeeding if mothers are educated about the need to avoid feeds other than breast milk and, if they are supported, to have faith that their milk is adequate for their babies, even if their health and nutritional status are less than ideal [44].

### CD4 count

Maternal CD4+ count has been used as an indicator to assess eligibility for antiretroviral treatment & prophylaxis for prevention of HIV-1 mother-to-child transmission and low CD4+ lymphocyte count was found associated with increased mother-to-child transmission risk [47-53]. Available studies have also shown that CD4 count increases with the use of triple-drug combination therapy [54].

### Hemoglobin

Studies conducted in Sub-Saharan Africa that have found low maternal hemoglobin level (<11 g/dl) during pregnancy, as a risk factor for HIV-1 mother- to-child transmission [55-57]. Anemia is a common clinical finding in HIV-infected patients. In these patients, many factors may contribute to the development of anemia, including nutritional deficiencies, opportunistic infections, AIDS-related malignancies, drug treatment and a direct effect of HIV on the bone marrow. Iron maldistribution may increase susceptibility to opportunistic infections and accelerate disease progression [57].

This study has some limitations. The self-reporting as an approach to provide survey data is likely to introduce specific biases for factors such as infant feeding practices, mutual disclosure of HIV status and individual socioeconomic characteristics. Although Muhima study findings corroborate those from existing literature about major risk factors for mother-to-child transmission of HIV-1 in breastfeeding populations, it was only conducted in one of the 30 Rwanda districts and located in urban area. For the results to be generalizable to the entire country there would be need for larger studies.

## Conclusion

In 679 mother – infant pairs followed at Muhima health Centre (Rwanda), the most relevant factors independently associated with increased risk of mother – to – child transmission of HIV-1 included non-disclosure of HIV status to partner and high HIV-1 RNA. Members of this cohort also showed socioeconomic inequalities, with unmarried status carrying higher risk of undisclosed HIV status that, as a mediator, was associated with higher risk of MTCT. Such findings suggest that HIV status disclosure to partner & HIV-1 RNA level are key entry points for reducing HIV-1 mother-to-child transmission in Rwanda. And more specifically, the monitoring of HIV-1 RNA level might be considered as a routinely used test to assess the risk of transmission with the goal of achieving viral suppression as critical for elimination of transmission, particularly in breastfeeding populations. In addition, further research is needed to identify most effective interventions to get optimal mutual disclosure of HIV status for Rwanda PMTCT services & clients.

## Abbreviations

AIDS: Acquired immunodeficiency syndrome; ARV: Antiretroviral; CD4: T-helper cells; CI: Confidence interval; DNA: Deoxyribonucleic Acid; HAART: Highly active antiretroviral therapy; HIV: Human immunodeficiency virus; OR: Odds ratio; PCR: Polymerase chain reaction; PMTCT: Prevention of mother-to-child transmission; RNA: Ribonucleic acid; WHO: World Health Organization

## Competing interests

Authors declare no competing interests.

## Authors’ contributions

MB supervised the study and wrote the paper. JDDB & JM assisted with data collection, entry and analysis. CPH contributed to the study design, data analysis and reviewed the manuscript critically for important intellectual content. All authors read and approved the final manuscript.
